# The proportion of dropouts from the maternity continuum of care and its predictors among antenatal booked women who gave birth in the last 12 months in Northwest Ethiopian women: a community-based cross-sectional study

**DOI:** 10.11604/pamj.2022.41.312.26298

**Published:** 2022-04-18

**Authors:** Nakachew Sewnet Amare, Bilen Mekonnen Araya, Mengstu Melkamu Asaye

**Affiliations:** 1 Clinical Midwifery, Department of Midwifery, College of Medicine and Health Sciences, Debre Berhan University, Debre Berhan, Ethiopia,; 2 Clinical Midwifery, School of Midwifery, College of Medicine and Health Sciences, University of Gondar, Gondar, Ethiopia,; 3 Women´s and Family Health Department, School of Midwifery, College of Medicine and Health Sciences, University of Gondar, Gondar, Ethiopia

**Keywords:** Dropout, Ethiopia, maternity, postnatal care, maternal health, continuum of care

## Abstract

**Introduction:**

maternity continuum of care is the continuity of maternity health care services that a woman uses in antenatal care, skill birth attendant, and postnatal care. This continuum of care in maternal health has become one of the government concerns and programs for planning and evaluating strategies within the currently existing maternal health system of Ethiopia. It is an important intervention in reducing maternal and neonatal morbidity and mortality. However, there is no clear information on the proportion of dropouts from the maternity continuum of care in Ethiopia. This study aimed to assess the proportion and associated factors of dropout from the maternity continuum of care among mothers who gave birth in the last 12 months in Debre Markos town, Northwest Ethiopia, 2018.

**Methods:**

a dropout from the maternity continuum of care was considered as a woman who had at least one visit of antenatal care but did not use SBA and postnatal care. A community-based cross-sectional study with a cluster sampling technique was conducted among 605 mothers who gave birth in the last 12 months in Debre Markos town. The data were collected from August 1-30/ 2018 using face-to-face interviews through pretested and semi-structured questionnaires. Multivariable logistic regression models were fitted to determine factors associated with dropout from the maternity continuum of care. P<0.05 was considered statistically significant.

**Results:**

the percentage of dropping out of the maternity continuum of care was found to be 32.2 % (95%CI: 28.4-36.2). No exposure to media (Adjusted Odds Ratio [AOR] = 2.62, CI: 1.47-4.68), women who heard about Postnatal care (AOR= 0.07, 95%CI: 0.04-0.15), unplanned pregnancy (AOR= 3.40, CI: 1.11-10.39), and having<4 Antenatal care follow up (AOR = 3.03, CI: 1.96-4.69) were statistically significant variables with the dropout from the maternity continuum of care.

**Conclusion:**

in this study, the proportion of dropouts from the maternity continuum of care is found to be high. Interventions aiming to improve retention in ANC care should be given emphasis.

## Introduction

The maternity continuum of care is defined as the continuity of maternity health care services that a woman uses antenatal care (ANC), skill birth attendant (SBA), and postnatal care (PNC) [1]. It is one of the important strategies for reducing maternal and newborn deaths and improving maternal and neonatal health and well-being [1, 2]. Providing appropriate maternal health care services in a continuum manner can reduce most of the preventable causes of maternal and neonatal death [3].

According to the World Health Organization (WHO), in 2015 estimation approximately 303,000 maternal deaths occurred globally, most of the deaths occur during labour, delivery, and the immediate post-partum period [2]. For subsequent reduction of this problem, the new globally adopted agenda between 2016 and 2030 as a part of the sustainable development goal aimed to reduce the maternal mortality ratio to 70 per 100,000 by addressing all maternal health care services for every woman as a top priority [4]. But women in most countries drop out before completing the full component of the maternity continuum of care [3]. In many countries, there is a significant dropout among women who had ANC visits before getting other subsequent maternal health care services [5, 6]. This dropout or failure to access skilled birth attendants and postnatal care results in compromised health and wellbeing of both the mother and newborn [7].

In Ethiopia, trends of reproductive health indicators from 2000 to 2014 showed that even though there is a significant improvement in maternal health care services utilization, the gap in the continuum of maternal health care services remains remarkably high [8]. According to Ethiopia Demographic and Health Survey (EDHS) 2016 report, out of all reproductive-aged women, 62% received antenatal care (ANC) and 28% had skilled delivery assistance, among women, who gave birth in the 2 years before the survey, 17% had a postnatal check [9]. This showed that there is a significant drop-out through the maternity continuum of care and still little progress has been made in closing the gap between the levels of maternal health care services. This default in the continuity of care constitutes missed opportunities and is a risk factor for poor maternal and child health outcomes. In Ethiopia, even though studies were carried out on the utilization of each component of the maternity continuum of care, no studies were conducted on dropouts from the maternity continuum of care to show the gap in between the level of continuity of maternal health care services. Due to this reason, there are limited interventions undertaken regarding dropouts from the maternity continuum of care.

**Objectives:** the study aimed to assess the proportion of dropouts from the maternity continuum of care and associated factors among mothers who gave birth in the last 12 months in Debre Markos town.

## Methods

**Study design:** a community-based cross-sectional study was conducted from August 1- 30, 2018.

**Setting:** this study was conducted in Debre Markos town, located in the East Gojjam zone of Amhara regional state, Northwest Ethiopia far from 299 km from Addis Ababa (the capital city of Ethiopia), and 265 km from Bahir Dar, the capital city of Amhara Regional state. According to the Population projection of Ethiopia for all regions at the woreda level from 2014 - to 2017, the total population of the town is estimated to be 92,470. Among these, 46,738 are females [10]. Currently, it has 7 kebeles which is the smallest administrative unit in Ethiopia. The total number of households within 7 kebeles currently is 5530. Debre Marcos town has one referral hospital, three public health centers, and seven private clinics that provide maternal health care services.

**Participants:** women who resided in the town for six months, who had at least one antenatal care visit during their recent pregnancy, who gave birth in the last 12 months in the town, and who had more than six weeks age of last birth assessed during the data collection period were included. On the other hand, women who were unable to communicate effectively and were seriously ill were exclusion criteria.

**Study size:** since there is no study conducted in Ethiopia, the Single population proportion formula was used by assuming 50% of the women who had at least one antenatal care visit dropout from the maternity continuum of care within a 5% margin of error and by assuming 1.5 design effect and 10% non-response rate the final required minimum sample size was estimated to be 634.

**Figure 1:**
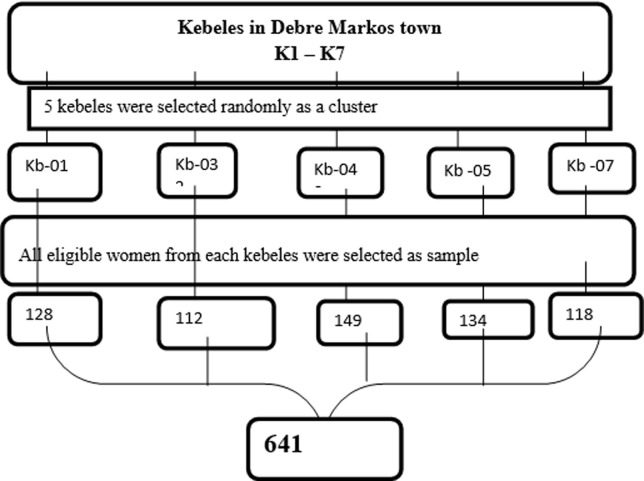
schematic presentation of the sampling procedure

**Sampling:** from seven kebeles (the smallest administrative units in the Ethiopian context) in the Town, we selected five kebeles randomly as a cluster. Then from each selected five clusters, we took all women who were eligible for the inclusion criteria finally, we attained 641 study participants from all selected clusters (Figure 1).

**Variables:** drop out from the maternity continuum of care was the outcome variable and others like socio-demographic characteristics, socio-cultural-related, husband and partner-related, reproductive obstetrics, and maternity health care service-related variables are explanatory variables included in the study. Exposure to media (the extent to which participants have TV or radio and encounter health-related news on it). In this study, dropout from the maternity continuum of care was considered as a woman who had at least one visit to ANC, but she did not use SBA and PNC [11-13]. The dropout at delivery (SBA) level was defined as a woman who had at least one ANC but did not use an SBA at the health institution during delivery [11-13]. A dropout at the PNC level was considered a woman who had at least one ANC but did not have PNC [11-13]. In this study, a woman who had PNC means If and only if the woman who gave birth at the health facility and revisited the facility to receive PNC [13].

**Data sources:** data were collected through a pre-tested semi-structured face-to-face interview. The semi-structured questionnaires were prepared in a local language, Amharic, to make them simple and understandable. Two diploma midwifery students for all selected kebeles for data collection and Bsc midwife supervisors, a total of two data collectors, and one supervisor were recruited. The questionnaire was prepared in English, and then translated to Amharic (local language) and back to English to maintain consistency of the tool. The training was provided for data collectors and supervisors for one day about the purpose of the study and techniques of data collection. The trained data collectors were supervised during data collection and each questionnaire was checked for completeness daily. Data entry was conducted by one computer. The questionnaire was pretested to check the response, language clarity, and appropriateness of the questionnaire while the pretest was done outside the study area at Basso lib-en woreda, Yejubie with 32 (5%) of the sample size. At the end of the pre-test depending on its outcome the correction measures like arrangements of questions were undertaken.

**Statistical methods:** first data were checked manually for completeness and then coded and entered into Epi Info version 7.1.2. Then data were exported to Statistical Package of Social Science (SPSS) version 20.00 for data checking cleaning (but we have no observations with missing data in this study), and analysis. Quantitative variables were summarized using the median and interquartile range (IQR) while categorical variables were described using frequencies and proportion. Bivariate logistic regression was used to identify independent variables associated with the maternity continuum of care. Independent variables having a p-value of less than 0.25 were entered into a multivariable logistic regression to adjust for confounding variables. Model fitness was checked using Hosmer and Lemeshow goodness of fit. Associations were described using an adjusted odds ratio (AOR) alongside their 95% confidence intervals (CI). A p-value <0.05 was considered statistically significant.

## Results

**Socio-demographic characteristics:** a total of 641 participants were found from the selected clusters for this study. Of these, 605 participants were interviewed, with a response rate of 94.4%. The median age of the participant was 28 years, with an interquartile range of 25 to 31 years. Five hundred sixty-nine (94%) of the women were orthodox by religion. Among respondents, 91.6% were married and 32.9% of them had attained tertiary education (college and above), whereas, 13.9% of them were never attended school. And 43% were housewives by occupation. The median monthly income of the respondents was 3000 ETB with an IQR of 1500-5000. The median age of the respondents' partners was 32 years with an IQR of 30 to 37rs. And 255 (42.1%) of participants' partners attained tertiary education (college and above). By occupation, 272 (45%) of institutions were private employees (Table 1).

**Table 1:** socio-demographic characteristics of study participants and their partners in Debre Markos town, Northwest, Ethiopia, January 2018 (n=605)

Characteristics	Frequency	Percentage
**Age of women in years**		
≤34	516	85.3
>34	89	14.7
**Age of partners in years**		
20-29	149	24.6
30-39	340	56.2
≥40	116	19.2
**Religion**		
Orthodox	569	94.1
Other* (Muslim, protestant, and catholic)	36	5.9
**Current marital status**		
Married	554	91.6
Other*(single, divorced, and separated)	51	8.4
**Educational status of women**		
Cannot read and write	84	13.9
Primary education(1-8)	128	21.2
Secondary education(9-12)	194	32.1
Tertiary(college and above)	199	32.9
**Educational status of the partner**		
Cannot read and write	49	8.1
Primary education(1-8)	98	16.2
Secondary education(9-12)	203	33.6
Tertiary(college and above)	255	42.1
**Occupational status of women**		
Housewife	260	43
Private employee	119	19.7
Gov`t employee	144	23.8
Other*(merchant, student, daily labor, and farmer)	82	13.5
**Average family monthly income**		
≤650 ETB	38	6.3
651-1610 ETB	142	23.5
≥1611 ETB	425	70.2
**Type of media they use**		
Television	499	82.5
Other*(television and newspaper)	77	12.7
**Occupational status of the partner**		
Private employee	272	45
Gov`t employee	220	36.4
Merchant	76	12.6
Other*(student, daily labor, and farmer)	37	6

* ETB=Ethiopian Birr

**Obstetrics and reproductive related characteristics:** among respondents, 9(1.5%) got services from traditional healers during pregnancy. Sixty-four (10.6%) of the women had four or more pregnancies. The majority (92.4%) of the participants' recent pregnancy was planned, and 581(96%) of the respondents' last pregnancy was wanted. (Table 2).

**Table 2:** obstetrics/reproductive related characteristics of study participants in Debre Markos town, Northwest, Ethiopia, January 2018 (n=605)

Characteristics	Frequency	Percentage
**Number of pregnancy**		
1	255	42.1
2	190	31.4
3	96	15.9
4 and above	64	10.6
**Was the pregnancy planned**		
Yes	559	92.4
No	46	7.6
**Was the pregnancy wanted**		
Yes	581	96
No	24	4
**Number of children**		
1	255	42.1
2	196	32.4
3	92	15.4
4 and above	62	10.2

**Maternity health care service-related characteristics:** of the respondents, 558(92.2%) heard about maternal health care services. And more than half of the study participants, (67.1%) never heard about postnatal care services. Most (85.5%) of the study participants start their first antenatal care follow-up at 1-3 months of pregnancy. Nearly half (47.9%) of the women in their ANC follow up were at the health center. Among respondents, 402(66.4%) of the women had four or more times ANC follow up during their recent pregnancy. Among the study participants, 509(84.1%) were getting birth to their last baby at the public hospital, and 1(0.2%) of the respondents delivered their last baby at home. Of those government or private employee women, 13% of them did not get permission from the workplace, and 11(1.8%) of the women also did not get permission from their husband or partner at the time they want to seek maternity care services (Table 3).

**Table 3:** maternity health care service-related characteristics of study participants in Debre Markos town, Northwest, Ethiopia, January 2018 (n=605)

Characteristics	Frequency	Percentage
**Ever heard about maternal health care services**		
Yes	558	92.2
No	47	7.8
**About ANC**		
Yes	555	91.7
No	50	8.3
**About delivery care**		
Yes	415	68.6
No	190	31.4
**About postnatal care**		
Yes	199	32.9
No	406	67.1
**Months of pregnancy at first ANC visit**		
1-3 month	517	85.5
4-7 month	88	14.5
**Place of ANC**		
Health center	290	47.9
Public hospital	274	45.3
Private health institution	81	13.4
**Number of ANC follow up**		
<4 times	203	33.6
≥4 times	402	66.4
**Place of delivery of the last baby**		
Health center	95	15.7
Public hospital	509	84.1
Home	1	0.2
**Distance to the health facility**		
0.5-4km	595	98.3
5-9km	10	1.7
**Getting permission from the workplace(n=298)**		
Yes	259	87
No	39	13
**Getting permission from husband/partner**		
Yes	594	98.2
No	11	1.8

**Bivariable and multivariable logistic regression analysis:** the percentage of dropouts from the maternity continuum of care was 32.2% (95% CI: 28.4-36.2). Those women who dropouts from the maternity continuum of care were never got PNC. On bivariable binary logistic regression, educational status of the partner, ever heard about ANC, ever heard about delivery care services, ever heard about PNC, exposure to media, having planned pregnancy, having wanted pregnancy, the total number of pregnancies and, number of ANC follow up had an association with drop out of maternity continuum of care at postnatal care level. The result of the multivariable analysis showed that ever heard about PNC, exposure to media, having a planned pregnancy and the number of ANC follow up were significantly associated with dropout from the maternity continuum of care at the postnatal care level. Those women who ever heard about PNC were 93% less likely to drop out of the maternity continuum of care compared to women who have not ever heard about PNC (AOR= 0.07, 95%CI: 0.04-0.15, P=0.0001). Of the study participants, those who did not use mass media were 2.62 times (AOR= 2.62, CI: 1.47-4.68, P=0.000) more likely to discontinue from the maternity continuum of care at the postnatal care level than women who use mass media. Respondents whose recent pregnancy was unplanned had 3.40 times (AOR= 3.40, CI: 1.11-10.39, P=0.03) more chance of discontinuing from the maternity continuum of care compared to women whose pregnancy was planned. Those women who had <4 ANC follow up during their last pregnancy were 3.03 times (AOR = 3.03, CI: 1.96-4.69, P=0.0001) more likely to drop out of the maternity continuum of care than women who had four and more ANC follow up throughout pregnancy (Table 4).

**Table 4:** bivariate and multivariable logistic regression analysis of factors associated with dropout from maternity continuum of care, in Debre Markos town, North West Ethiopia, 2018(n=605)

Variables	Drop out from maternity continuum of care		COR (95%CI)		AOR (95%CI)
	Yes	No			
**Maternal education**					
Cannot read and write	31	18	7.43 (3.84-14.37)		1.41 (0.62-3.23)
Primary education(1-8)	43	55	3.37 (2.03-5.60)		0.91 (0.48-1.71)
Secondary education(9-12)	73	130	2.42 (1.58-3.71)		1.26 (0.76-2.08)
Tertiary(college and above)	48	207	1		
**Ever heard about ANC**					
No	27	23	2.70 (1.51-4.85)		0.72 (0.35-1.49)
Yes	168	387	1		
**Ever heard about DC**					
No	98	92	3.49 (2.43-5.026)		1.43 (0.88-2.34)
Yes	97	318	1		
**Ever heard about PNC**					
No	186	220	1		
Yes	9	190	0.06 (0.03-0.11)		0.07 (0.04-0.15)**
**Exposure to media**					
No	62	29	6.12 (3.78-9.93)		2.62 (1.47-4.68)**
Yes	133	381	1		
**Was the last pregnancy planned?**					
No	28	18	3.65 (1.96-6.78)		3.40 (1.11-10.39)0.03
Yes	167	392	1		
**Was last pregnancy wanted?**					
No	12	12	2.18 (0.96-4.93)		0.32 (0.08-1.40)
Yes	183	398	1		
**Number of ANC follow up**					
<4	104	99	3.59 (2.50-5.15)		3.03 (1.963-4.685) **
≥ 4	91	311	1		
**Total number of pregnancy**					
1	88	167	1		
2	47	143	0.62 (0.41-0.95)		0.74 (0.45-1.22)
3	32	64	0.95 (0.58-1.56)		1.38 (0.73-2.59)
4+	28	36	1.48 (0.85-2.58)		1.68 (0.85-3.34)

ANC=Antenatal Care, DC= Delivery Care, PNC= Post Natal Care, COR= Crude odds ratio, AOR=Adjusted odds ratio, CI=Confidence interval, 1=reference category. ** P ≤0.001

## Discussion

The study found that the overall dropout from the maternity continuum of care was 32.2%. Compared to dropping out and never got skilled birth attendance the much higher drop out was found at the postnatal care level, that was 32.2% which is the same as the overall drop out, This indicates, those women who delivered at health institutions and after discharged they never visit health institution to seek postnatal care and the women who delivered at home never visit the health institution for PNC within 6 weeks of post-partum.

The drop-out between ANC and delivery care of 0.2 % was much lower than the results of the studies conducted in Nigeria (38.1%) [11], Uganda (42%) [3], and Cambodia (21%) [14]. The possible difference observed from a study in Uganda might be because there is a socio-cultural and gender issue in Uganda that prohibits women to give birth in health institutions, such as preferred birthing positions and more preference for traditional birth attendants [3]. This might result in a higher dropout between ANC and SBA than our findings. But, there was no study found in line with or lower than the finding of our study. Regarding postnatal care dropouts in this study, 32.2% were lower compared to the study carried out in Nigeria (50.8%) [11]. This variation is possibly due to this study being conducted among all Nigerian women giving birth 5 years before data collection from nationally representative data of NDHS 2013. This nationwide study includes women from a rural part of the country where relatively had limited opportunities to get postnatal care or all maternity care services than in urban, which results in a relatively higher dropout of women from the maternity continuum of care at the postnatal care level than our study since our study is conducted among the women who reside in the town.

On the other hand, the dropout of PNC in this study was higher than in the study conducted in Cambodia (25%) [14]. The reason for this variation might be those studies in Cambodia were considered postnatal care drop out if the women did not receive postnatal care services within the first 48 hours after delivery. This may lower the finding because most of the women delivered in the health institution stay for at least 6 hours until discharge, so most of them counted as they had postnatal care. But, in our study to say a woman had postnatal care if she gave birth in the health institution and after discharge, a woman should return to or visit the health institution to seek postnatal care services.

In this study, participants who did not use mass media were 2.62 times more likely to discontinue before getting PNC services than women who use mass media. This finding was supported by different studies conducted in Holeta town, central Ethiopia [15], Hawassa, Ethiopia [16], Pakistan [17], Bangladesh [18], Nepal [12]. This may be due to the reason that, even though the women had at least one ANC follow up, mass media is important to promote information about maternal health and the importance of seeking maternity care services in a continuum manner; this helps the women to improve their knowledge and attitude towards maternity continuum of care. But, women who didn't use mass media might not be able to access such information, and this leads to the women dropping out of the maternity continuum of care.

The analysis of this study showed that women who ever heard about PNC services were 93% less likely to drop out of the maternity continuum of care at the postnatal care level than those women who didn't even hear about it. Similar findings were reported by the study done in Indonesia [19]. This is possible because women who had information about postnatal care might know about the post-partum period which is the time for the majority of maternal death and it is also the most critical time for maternal survival by receiving proper postnatal care services, and they also know the paramount of getting all level of maternal health care services. This leads to reducing the dropout from the maternity continuum of care at the postnatal care level.

This study also revealed that women who had unplanned pregnancies were 3.40 times more likely to drop out from the maternity continuum of care at the postnatal care level than those women who had timed or planned pregnancies. This finding is supported by the study conducted on PNC utilization in Debre Tabor town, Northwest Ethiopia [20], Turkey [21]. The possible explanation might be due to the unplanned or mistimed pregnancy precluding the chance of getting proper care or more likely to delay initiation of care during pregnancy, and this results in the women having less preparation for parenthood and having less adequate information regarding the importance of getting maternity continuum of care.

Our study also suggested the significant associations between having fewer than 4 ANC follow up with the proportion of postnatal care drop out, study participants who had fewer than 4 ANC follow up during their recent pregnancy were 3.03 times more likely to drop out before getting PNC services, than those women who had four and more ANC. Similar findings on the study conducted in Nigeria [22], Cambodia [14], Nepal [12]. This might be since this is connected with accessing SBA at the delivery level and postnatal care, having ANC follow up especially for and more ANC follow up is important to have time to get information's about the importance of giving birth in the health institution, this opportunity also helps the women to get knowledge about postnatal care schedules, important services that the women will get on those schedules and the importance of getting those services. Compared to having fewer than 4 ANC follow up, having 4 and more ANC had its role in hindering drop-out before seeking postnatal care services as well drop-out from the maternity continuum of care.

However, in our study, religion, educational status of the women and partner, number of pregnancies, occupational status, exposure to witchcraft, and distance to health facility had not a significant association with the dropout from the maternity continuum of care. Studies revealed that distance to the health facility [11], having more than four pregnancies [12], and exposure to witchcraft [23], had an effect by increasing the likelihood of women dropping out of the maternity continuum of care.

**Limitation of the study:** we could not ascertain the causality link between the outcome and the exposures. Because both are examined at the same time. There might be recall and reporting bias by the study participants regarding events from the past. We tried to decrease this bias through the careful organization of the questionnaires. In addition, there might be residual confounding from measured and unmeasured confounders.

## Conclusion

The proportion of drop out from the maternity continuum of care was found to be high. All those women who had dropout from the maternity continuum of care had a dropout at the postnatal care level. In this study; no exposure to media, women ever heard about PNC, unplanned pregnancy, and having fewer than 4 ANC follow up were determinants of dropout from the maternity continuum of care.

### What is known about this topic

- The utilization of institutional delivery services is low;
- Husband involvement in maternal service utilization is low.

### What this study adds

- Dropout from postnatal care was high;
- Having less than four ANC follow up was a contributing factor to high dropout;
- The utilization of institutional delivery was high.
